# Maternal cigarette smoking before or during pregnancy increases the risk of birth congenital anomalies: a population-based retrospective cohort study of 12 million mother-infant pairs

**DOI:** 10.1186/s12916-021-02196-x

**Published:** 2022-01-11

**Authors:** Lili Yang, Huan Wang, Liu Yang, Min Zhao, Yajun Guo, Pascal Bovet, Bo Xi

**Affiliations:** 1 Department of Epidemiology, School of Public Health, Qilu Hospital, Cheeloo College of Medicine, Shandong University, Jinan, China; 2 Department of Nutrition and Food Hygiene, School of Public Health, Cheeloo College of Medicine, Shandong University, Jinan, China; 3 Department of Epidemiology, School of Public Health and Tropical Medicine, Tulane University, New Orleans, USA; 4 Center for Primary Care and Public Health (Unisanté), University of Lausanne, Lausanne, Switzerland

**Keywords:** Cigarette smoking, Congenital anomalies, Pregnancy, Offspring

## Abstract

**Background:**

The associations of maternal cigarette smoking with congenital anomalies in offspring have been inconsistent. This study aimed to clarify the associations of the timing and intensity of maternal cigarette smoking with 12 subtypes of birth congenital anomalies based on a nationwide large birth cohort in the USA.

**Methods:**

We used nationwide birth certificate data from the US National Vital Statistics System during 2016–2019. Women reported the average daily number of cigarettes they consumed 3 months before pregnancy and in each subsequent trimester during pregnancy. Twelve subtypes of congenital anomalies were identified in medical records. Poisson regression analysis was used to estimate the risk ratios (RRs) with 95% confidence intervals (CIs) for 12 subtypes of congenital anomalies associated with the timing (i.e., before pregnancy, and during three different trimesters of pregnancy) and intensity (i.e., number of cigarettes consumed per day) of maternal cigarette smoking.

**Results:**

Among the 12,144,972 women included, 9.3% smoked before pregnancy and 7.0%, 6.0%, and 5.7% in the first, second, and third trimester, respectively. Maternal smoking before or during pregnancy significantly increased the risk of six subtypes of birth congenital anomalies (i.e., congenital diaphragmatic hernia, gastroschisis, limb reduction defect, cleft lip with or without cleft palate, cleft palate alone, and hypospadias), even as low as 1–5 cigarettes per day. The adjusted RRs (95% CIs) for overall birth congenital anomalies (defined as having any one of the congenital malformations above significantly associated with maternal cigarette smoking) among women who smoked 1–5, 6–10, and ≥ 11 cigarettes per day before pregnancy were 1.31 (1.22–1.41), 1.25 (1.17–1.33), and 1.35 (1.28–1.43), respectively. Corresponding values were 1.23 (1.14–1.33), 1.33 (1.24–1.42), 1.33 (1.23–1.43), respectively, for women who smoked cigarettes in the first trimester; 1.32 (1.21–1.44), 1.36 (1.26–1.47), and 1.38 (1.23–1.54), respectively, for women who smoked cigarettes in the second trimester; and 1.33 (1.22–1.44), 1.35 (1.24–1.47), and 1.35 (1.19–1.52), respectively, for women who smoked cigarettes in the third trimester. Compared with women who kept smoking before and throughout pregnancy, women who never smoked had significantly lower risk of congenital anomalies (RR 0.77, 95% CI 0.73–0.81), but women who smoked before pregnancy and quitted during each trimester of pregnancy had no reduced risk (all *P* > 0.05).

**Conclusions:**

Maternal smoking before or during pregnancy increased the risk of several birth congenital anomalies, even as low as 1–5 cigarettes per day. Maternal smokers who stopped smoking in the subsequent trimesters of pregnancy were still at an increased risk of birth congenital anomalies. Our findings highlighted that smoking cessation interventions should be implemented before pregnancy.

**Supplementary Information:**

The online version contains supplementary material available at 10.1186/s12916-021-02196-x.

## Background

Congenital anomalies, also known as congenital malformations or birth defects, can be defined as structural or functional anomalies that are diagnosed prenatally or after delivery [1]. Globally, approximately 8 million infants (6% of total births) are estimated to be born with a serious congenital anomaly every year [2]. In the USA, congenital anomalies are the leading cause of infant mortality, accounting for 21% of infant deaths in 2017 [3]. The total hospital charges for treating congenital anomalies exceed $2.6 billion each year in the USA [4]. Thus, identifying modifiable risk factors of congenital anomalies and implementing effective primary prevention strategies remain a priority of public health policies in the USA and worldwide [1, 3].

In the USA, nearly 16.0% of women aged 25–44 years were current cigarette smokers in 2015 [5], and at least half of them continued smoking during pregnancy [6, 7]. It means that approximately 360,000 infants born in the USA were exposed to cigarette smoking [8]. Maternal cigarette smoking during pregnancy is associated with many detrimental infant health outcomes, such as delayed intrauterine development, preterm birth, and infant mortality [9–11]. However, the associations between maternal cigarette smoking during pregnancy and congenital anomalies remain less clear. Several studies reported that maternal cigarette smoking was significantly associated with an increased risk of certain type of congenital anomalies, such as oral clefts [12, 13] and congenital cardiovascular malformations [13, 14]. In contrast, other studies showed no association between maternal cigarette smoking and any type of congenital anomalies [15, 16]. Moreover, a few studies even reported a decreased risk of certain specific anomalies among women who smoked in pregnancy, such as anomalies on musculoskeletal system [13], and hypospadias [17]. These previous studies [12–17] had several limitations such as the inclusion of relatively small number of cases, especially for rare type of congenital anomalies (i.e., being underpowered to detect small or moderate risk effect), the use of case-control design (i.e., being more prone to selection bias), or insufficient adjustment for potential confounders (i.e., being more prone to confounding bias). In terms of smoking intensity, the associations of maternal cigarette smoking and congenital anomalies in offspring were also inconsistent. For example, a case-control study in the USA showed that light smoking (< 10 cigarettes per day) significantly increased the risk of cleft lip with or without cleft palate (odds ratio [OR] 1.50, 95% CI 1.28–1.76) [18]. Another case-control study in the UK reported that smoking 1–10 cigarettes per day during pregnancy marginally increased the risk of this anomaly (OR 1.70, 95% CI 0.90–3.90) [12]. In contrast, a large population-based prospective cohort study in Denmark showed that light smoking (≤ 5 cigarettes per day) was associated with a marginally reduced risk of major malformations (OR 0.96, 95% CI 0.92–1.01) [13]. These inconsistent results can obscure the teratogenic effects of maternal cigarette smoking during pregnancy to public policy makers and weaken tobacco control efforts aiming at preventing congenital anomalies. In addition, the previous studies mentioned above [12–17] did not distinguish the effect of different maternal smoking timing (before or during different trimesters of pregnancy) on congenital anomalies, and little is known about whether and when smoking cessation during pregnancy matter for birth congenital anomalies.

Based on a nationwide large birth cohort data in the USA (*N* = 12,144,972), this study was aimed to examine the associations of the timing (before pregnancy, and during different trimesters of pregnancy) and intensity (number of cigarettes consumed per day) of maternal cigarette smoking with 12 subtypes of birth congenital anomalies.

## Methods

### Study population

This study used nationwide birth certificate data from the National Vital Statistics System (NVSS) in the USA (2016-2019). The NVSS is a joint program led by the US Centers for Disease Control and Prevention and all US states. It collected data on a wide range of maternal and infant demographic and health information. Standardized questionnaires and registration procedures have been developed and recommended for all states and the District of Columbia in the USA. Two worksheets have been developed since 2003, including the mother’s worksheet and the facility worksheet. The mother`s worksheet compiles mother data around the time of delivery (e.g., maternal demographics, and cigarette smoking before and during pregnancy). The facility worksheet compiles other data appearing in medical records (e.g., risk factors in this pregnancy, and birth congenital anomalies). More details about NVSS data collection can be found at the official website (https:// www.cdc.gov/nchs/nvss/births.htm). The birth certificate data from the NVSS are de-identified and do not include any protected health information. The data are publically available [19] and exempt under the ethical board review of the corresponding author’s institution.

**Fig. 1:**
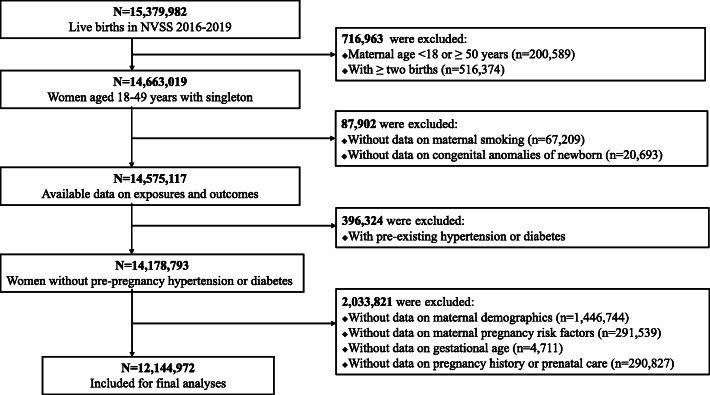
Flow chart of the inclusion/exclusion of the participants

In this study, we used NVSS data from 2016 to 2019 because all US states and the District of Columbia had completely implemented the 2003 version of Standard Certificate of Live Birth to collect birth information since 2016. A total of 15,379,982 live births registered in the NVSS from 2016 to 2019 were preliminarily included in the study. We then excluded women aged < 18 or ≥ 50 years (*n* = 200,589) in order to only include adult women of reproductive age, women with twin or multiple births (*n* = 516,374) because they differed from the singletons with congenital anomalies [20], and women with pre-existing hypertension or diabetes (*n* = 396,324) because these chronic conditions were strong risk factors for birth congenital anomalies [21, 22]. We further excluded women with missing data on maternal cigarette smoking (*n* = 67,209), birth congenital anomalies (*n* = 20,693), or on covariates (maternal demographics, maternal pregnancy risk factors, gestational age at delivery, and pregnancy history or prenatal care; *n* = 2,033,821). This left a total of 12,144,972 live singleton births included for the final data analysis. Figure 1 shows the flow chart of the inclusion/exclusion of the participants.

### Maternal cigarette smoking

Information on maternal cigarette smoking was collected using the mother’s worksheet in the hospital around the time of delivery. Mothers reported the average daily number of cigarettes consumed 3 months prior to pregnancy and during the first, second, and third trimesters of pregnancy. In each time period, women who smoked one or more cigarettes per day were considered as smokers, whereas women who smoked “0” cigarette per day were considered as non-smokers. Smokers were further categorized into three smoking intensity subgroups: 1–5, 6–10, and ≥ 11 cigarettes per day. These categories were defined in order to warrant robust statistical power in each subgroup and also were based on previous studies [13].

### Congenital anomalies

Information on birth congenital anomalies was obtained from the facility worksheet. Twelve subtypes of congenital anomalies were identified at birth, including anencephaly, meningomyelocele/spina bifida, cyanotic congenital heart disease, congenital diaphragmatic hernia, omphalocele, gastroschisis, limb reduction defect, cleft lip with or without cleft palate, cleft palate alone, Down syndrome, suspected chromosomal disorder, and hypospadias. For hypospadias, data were restricted to male infants. For Down syndrome and other suspected chromosomal disorders, we combined results of the pending and confirmed karyotype. In addition, we defined “overall congenital anomalies” as having any type of congenital malformations above that were significantly associated with maternal cigarette smoking.

### Study covariates

Data on maternal demographics, educational level, marital status, and maternal pre-pregnancy weight and height were collected using the mother’s worksheet. Data on maternal risk factors (i.e. eclampsia, gestational hypertension and gestational diabetes), parity, infant sex, gestational age at delivery, and prenatal care visits were obtained from the facility worksheet.

We categorized covariates as follows: maternal age at delivery (18–29, 30–39, or 40–49 years), race/ethnicity (Hispanic, non-Hispanic white, non-Hispanic black, or other), educational level (less than high school, high school, or more than high school), marital status (married or unmarried), maternal pre-pregnancy body mass index (BMI) (< 18.5, 18.5–24.9, 25.0–29.9, 30.0–34.9, 35.0–39.9, or ≥ 40 kg/m^2^ based on the World Health Organization guidelines [23]), eclampsia (yes or no), gestational hypertension (yes or no), gestational diabetes (yes or no), parity (1, 2, 3, or ≥ 4), infant sex (male or female), and total number of prenatal care visits (0, 1–4, 5–9, or ≥ 10).

### Statistical analysis

Baseline characteristics of the participants were presented according to maternal cigarette smoking status before pregnancy and in the first, second, and third pregnancy trimesters. Comparisons between groups were analyzed using *χ*^2^ test for categorical variables and Wilcoxon test for continuous variables. We first used Poisson regression analysis to estimate the associations of maternal cigarette smoking status (yes versus no) before or during pregnancy with each of 12 subtypes of congenital anomalies using women who did not smoke cigarettes in the corresponding period as the reference. We adjusted for several covariates, including maternal age at delivery, race/ethnicity, educational level, marital status, maternal pre-pregnancy BMI, eclampsia, gestational hypertension, gestational diabetes, parity, infant sex, gestational age at delivery, and total number of prenatal care visits. These potential confounders were selected based on the literature (i.e., based on their known/hypothetic correlations with both the exposures [i.e., maternal smoking] and outcomes [i.e., congenital anomalies]) as well as the information that was available on the birth certificate. We then estimated the risk ratios (RRs) and 95% confidence intervals (CIs) of birth congenital anomalies (i.e., which showed positive associations in the initial analysis) according to smoking intensity before and during each trimester of pregnancy. We also examined the association of smoking cessation in different trimesters of pregnancy with overall birth congenital anomalies using women who kept smoking before and throughout pregnancy as the reference. We performed four sensitivity analyses to test the stability of our main findings (i.e., the association between the timing of maternal cigarette smoking and six subtypes of congenital anomalies), including the exclusion of women with cesarean section, the exclusion of those with eclampsia, gestational hypertension or gestational diabetes, the exclusion of those with preterm delivery, and the use of imputation analyses (using the fully conditional specification method [24]) to deal with missing data on all variables of interest. All statistical analyses were performed using SAS V.9.4 (SAS Institute Inc., Cary, North Carolina). A two-tailed *p* < 0.05 was considered statistically significant. The Bonferroni adjustment method was used for multiple comparisons.

## Results

### Population characteristics

A total of 12,144,972 women with live singleton births were included, with a mean age at delivery of 29 years (interquartile range 25–33 years). 9.3% of the women smoked cigarettes before pregnancy, respectively 7.0%, 6.0%, and 5.7% in the first, second, and third trimesters. In general, women who smoked cigarettes before or during pregnancy tended to be younger and non-Hispanic white, to have a low educational level, to be unmarried, obese, and multiparous (all *p* < 0.0001). Detailed characteristics of the study population are shown in Table 1.

**Table 1:** Characteristics of the study population

	Total	Smoking before pregnancy			Smoking in the first trimester			Smoking in the second trimester			Smoking in the third trimester		
		No	Yes	*P* value*	No	Yes	*P* value*	No	Yes	*P* value*	No	Yes	*P* value*
***N***	12144972	11011647	1130017		11295453	846288		11417309	724008		11433236	687173	
**Maternal age at delivery, years**													
Median (interquartile range)	29 (25–33)	29 (25–33)	27 (23–31)	< 0.0001	29 (25–33)	27 (23–31)	< 0.0001	29 (25–33)	27 (23–31)	< 0.0001	29 (25–33)	27 (23–31)	< 0.0001
**Category,** ***n*** **(%)**				< 0.0001			< 0.0001			< 0.0001			< 0.0001
18–29	6633206 (54.6)	5853770 (53.2)	777222 (68.8)		6051459 (53.6)	579567 (68.5)		6139155 (53.8)	491571 (67.9)		6153808 (53.8)	465025 (67.7)	
30–39	5158070 (42.5)	4821115 (43.8)	335908 (29.7)		4903379 (43.4)	253694 (30.0)		4935865 (43.2)	221082 (30.5)		4937444 (43.2)	211273 (30.8)	
40–49	353696 (2.9)	336762 (3.1)	16887 (1.5)		340615 (3.0)	13027 (1.5)		342289 (3.0)	11355 (1.6)		341984 (3.0)	10875 (1.6)	
**Race/ethnicity,** ***n*** **(%)**				< 0.0001			< 0.0001			< 0.0001			< 0.0001
Hispanic	2594603 (21.4)	2515412 (22.8)	78738 (7.0)		2546720 (22.6)	47554 (5.6)		2557944 (22.4)	36268 (5.0)		2556280 (22.4)	33474 (4.9)	
Non-Hispanic white	6702736 (55.2)	5843582 (53.1)	857114 (75.9)		6044060 (53.5)	656548 (77.6)		6129954 (53.7)	570408 (78.8)		6149206 (53.8)	543958 (79.2)	
Non-Hispanic black	1773802 (14.6)	1648063 (15.0)	125290 (11.1)		1681172 (14.9)	92231 (10.9)		1697196 (14.9)	76148 (10.5)		1694668 (14.8)	71043 (10.3)	
Other	1073831 (8.8)	1004590 (9.1)	68875 (6.1)		1023501 (9.1)	49955 (5.9)		1032215 (9.0)	41184 (5.7)		1033082 (9.0)	38698 (5.6)	
**Education level,** ***n*** **(%)**				< 0.0001			< 0.0001			< 0.0001			< 0.0001
Less than high school	1446923 (11.9)	1212753 (11.0)	233557 (20.7)		1246992 (11.0)	199297 (23.6)		1265771 (11.1)	180425 (24.9)		1269412 (11.1)	173981 (25.3)	
High school	3138189 (25.8)	2665352 (24.2)	471540 (41.7)		2770947 (24.5)	366018 (43.3)		2820303 (24.7)	316486 (43.7)		2828893 (24.7)	300653 (43.8)	
More than high school	7559860 (62.3)	7133542 (64.8)	424920 (37.6)		7277514 (64.4)	280973 (33.2)		7331235 (64.2)	227097 (31.4)		7334931 (64.2)	212539 (30.9)	
**Marital status,** ***n*** **(%)**				< 0.0001			< 0.0001			< 0.0001			< 0.0001
Married	7413803 (61.0)	7077878 (64.3)	334872(29.6)		7183325 (63.6)	229352 (27.1)		7217956 (63.2)	194619 (26.9)		7217754 (63.1)	185756 (27.0)	
Unmarried	4731169 (39.0)	3933769 (35.7)	795145(70.4)		4112128 (36.4)	616936 (72.9)		4199353 (36.8)	529389 (73.1)		4215482 (36.9)	501417 (73.0)	
**Maternal pre-pregnancy BMI,** ***n*** **(%)**				< 0.0001			< 0.0001			< 0.0001			< 0.0001
< 18.5	398617 (3.3)	338266 (3.1)	60219 (5.3)		349638 (3.1)	48847 (5.8)		355457 (3.1)	43016 (5.9)		356831 (3.1)	40934 (6.0)	
18.5–24.9	5258272 (43.3)	4808587 (43.7)	448405 (39.7)		4915778 (43.5)	341196 (40.3)		4962552 (43.5)	294233 (40.6)		4970360 (43.5)	279659 (40.7)	
25.0–29.9	3226439 (26.6)	2947167 (26.8)	278406 (24.6)		3020845 (26.7)	204767 (24.2)		3051195 (26.7)	174315 (24.1)		3054557 (26.7)	165555 (24.1)	
30.0–-34.9	1793991 (14.8)	1615555 (14.7)	177896 (15.7)		1663012 (14.7)	130475 (15.4)		1683067 (14.7)	110357 (15.2)		1685008 (14.7)	104585 (15.2)	
35.0–39.9	869844 (7.2)	774057 (7.0)	95506 (8.5)		799561 (7.1)	70016 (8.3)		810359 (7.1)	59176 (8.2)		811208 (7.1)	55947 (8.1)	
≥ 40	597809 (4.9)	528015 (4.8)	69585 (6.2)		546619 (4.8)	50987 (6.0)		554679 (4.9)	42911 (5.9)		555272 (4.9)	40493 (5.9)	
**Eclampsia,** ***n*** **(%)**	30111 (0.3)	27230 (0.3)	2873 (0.3)	0.1565	28016 (0.3)	2076 (0.3)	0.6271	28342 (0.3)	1746 (0.2)	0.2400	28271 (0.3)	1624 (0.2)	0.0758
**Gestational hypertension,** ***n*** **(%)**	828884 (6.8)	747722 (6.8)	80863 (7.2)	< 0.0001	771842 (6.8)	56769 (6.7)	< 0.0001	782007 (6.9)	46563 (6.4)	< 0.0001	783133 (6.9)	43423 (6.3)	< 0.0001
**Gestational diabetes,** ***n*** **(%)**	766668 (6.3)	697156 (6.3)	69284 (6.1)	< 0.0001	717261 (6.4)	49195 (5.8)	< 0.0001	725557 (6.4)	40868 (5.6)	< 0.0001	727192 (6.4)	38653 (5.6)	< 0.0001
**Parity,** ***n*** **(%)**				< 0.0001	<0.0001 <0.0001 <0.0001								
1	4604618 (37.9)	4226115 (38.4)	377146 (33.4)		4359594 (38.6)	243802 (28.8)		4412457 (38.7)	190769 (26.4)		4416750 (38.6)	176170 (25.6)	
2	3941322 (32.5)	3605704 (32.7)	334684 (29.6)		3688377 (32.7)	251985 (29.8)		3723521 (32.6)	216701 (29.9)		3729275 (32.6)	205941 (30.0)	
3	2089008 (17.2)	1864709 (16.9)	223754 (19.8)		1906643 (16.9)	181789 (21.5)		1926676 (16.9)	161710 (22.3)		1930274 (16.9)	155298 (22.6)	
≥4	1510024 (12.4)	1315119 (11.9)	194433 (17.2)		1340839 (11.9)	168712 (19.9)		1354655 (11.9)	154828 (21.4)		1356937 (11.9)	149764 (21.8)	
**Infant sex,** ***n*** **(%)**				0.4854			0.3737			0.1427			0.2976
Male	6212985 (51.2)	5633535 (51.2)	577725 (51.1)		5777992 (51.2)	433328 (51.2)		5840111 (51.2)	370983 (51.2)		5847928 (51.2)	351923 (51.2)	
Female	5931987 (48.8)	5378112 (48.8)	552292 (48.9)		5517461 (48.9)	412960 (48.8)		5577198 (48.9)	353025 (48.8)		5585308 (48.9)	335250 (48.8)	
**Gestational age at delivery, weeks, median (interquartile range)**	39 (38–40)	39 (38–40)	39 (38–39)	< 0.0001	39 (38–40)	39 (38–39)	< 0.0001	39 (38–40)	39 (38–39)	< 0.0001	39 (38–40)	39 (38–39)	< 0.0001
**Total number of prenatal care visits,** ***n*** **(%)**				< 0.0001			< 0.0001			< 0.0001			< 0.0001
0	192355 (1.6)	157685 (1.4)	34613 (3.1)		160767 (1.4)	31511 (3.7)		162638 (1.4)	29629 (4.1)		161541 (1.4)	28498 (4.2)	
1–4	453702 (3.7)	374231 (3.4)	79251 (7.0)		384157 (3.4)	69312 (8.2)		390034 (3.4)	63413 (8.8)		385657 (3.4)	60258 (8.8)	
5–9	2511582 (20.7)	2230669 (20.3)	280034 (24.8)		2287074 (20.3)	223700 (26.4)		2314457 (20.3)	196206 (27.1)		2315802 (20.3)	186173 (27.1)	
≥ 10	8987333 (74.0)	8249062 (74.9)	736119 (65.1)		8463455 (74.9)	521765 (61.7)		8550180 (74.9)	434760 (60.1)		8570236 (75.0)	412244 (60.0)	

*BMI* body mass index

*Differences between groups were tested by Wilcoxon test for continuous variables, and chi-square test for categorical variables

### Associations between the timing of maternal cigarette smoking and birth congenital anomalies

Maternal cigarette smoking before pregnancy or during either trimester was significantly associated with six subtypes of congenital anomalies: congenital diaphragmatic hernia, gastroschisis, limb reduction defect, cleft lip with or without cleft palate, cleft palate alone, and hypospadias. For example, the adjusted RRs (95% CIs) of cleft lip with or without cleft palate of infants born to women who smoked cigarettes before pregnancy, and in the first, second, and third trimesters were 1.28 (1.18–1.38), 1.26 (1.16–1.37), 1.26 (1.15–1.38), and 1.24 (1.13–1.36), respectively (all *p* < 0.0001) (Table 2). Associations withstood multiple comparison correction, except for congenital diaphragmatic hernia and hypospadias during pregnancy.

**Table 2:** Associations of the timing of maternal cigarette smoking with birth congenital anomalies

Outcomes	Smoking before pregnancy			Smoking in the first trimester			Smoking in the second trimester			Smoking in the third trimester	
	No	Yes		No	Yes		No	Yes		No	Yes
**Gastroschisis**											
*n* (/10000)	2116 (1.92)	604 (5.35)		2263 (2.00)	457 (5.40)		2332 (2.04)	388 (5.36)		2350 (2.06)	358 (5.21)
RR (95% CI)	1.00	1.54 (1.40–1.70)		1.00	1.44 (1.29–1.61)		1.00	1.41 (1.25–1.58)		1.00	1.42 (1.26–1.61)
*P* value		< 0.0001			< 0.0001			< 0.0001			< 0.0001
**Limb reduction defect**											
*n* (/10000)	1384 (1.26)	272 (2.41)		1433 (1.27)	222 (2.62)		1468 (1.29)	187 (2.58)		1474 (1.29)	177 (2.58)
RR (95% CI)	1.00	1.52 (1.31–1.75)		1.00	1.58 (1.35–1.84)		1.00	1.49 (1.27–1.76)		1.00	1.49 (1.26–1.76)
*P* value		< 0.0001			< 0.0001			< 0.0001			< 0.0001
**Cleft palate alone**											
*n* (/10000)	2479 (2.25)	489 (4.33)		2554 (2.26)	413 (4.88)		2624 (2.30)	342 (4.72)		2630 (2.30)	330 (4.80)
RR (95% CI)	1.00	1.45 (1.30–1.61)		1.00	1.60 (1.43–1.79)		1.00	1.48 (1.31–1.67)		1.00	1.50 (1.33–1.70)
*P* value		< 0.0001			< 0.0001			< 0.0001			< 0.0001
**Cleft lip with or without cleft palate**											
*n* (/10000)	5462 (4.96)	884 (7.82)		5660 (5.01)	685 (8.09)		5747 (5.03)	597 (8.25)		5762 (5.04)	560 (8.15)
RR (95% CI)	1.00	1.28 (1.18–1.38)		1.00	1.26 (1.16–1.37)		1.00	1.26 (1.15–1.38)		1.00	1.24 (1.13–1.36)
*P* value		< 0.0001			< 0.0001			< 0.0001			< 0.0001
**Congenital diaphragmatic hernia**											
*n* (/10000)	1263 (1.15)	205 (1.81)		1314 (1.16)	154 (1.82)		1327 (1.16)	140 (1.93)		1325 (1.16)	130 (1.89)
RR (95% CI)	1.00	1.35 (1.15–1.58)		1.00	1.28 (1.07–1.53)		1.00	1.35 (1.12–1.63)		1.00	1.33 (1.10–1.62)
*P* value		0.0002			0.0072			0.0016			0.0036
Hypospadias*											
*n* (/10000)	6680 (11.86)	875 (15.15)		6910 (11.96)	646 (14.91)		7011 (12.00)	543 (14.64)		7021 (12.01)	517 (14.69)
RR (95% CI)	1.00	1.15 (1.07–1.24)		1.00	1.12 (1.03–1.22)		1.00	1.09 (1.00–1.20)		1.00	1.10 (1.00–1.21)
*P* value		0.0003			0.0089			0.0557			0.0460
**Omphalocele**											
*n* (/10000)	1045 (0.95)	163 (1.44)		1086 (0.96)	122 (1.44)		1105 (0.97)	103 (1.42)		1089 (0.95)	97 (1.41)
RR (95% CI)	1.00	1.22 (1.02–1.45)		1.00	1.15 (0.94–1.41)		1.00	1.12 (0.91–1.39)		1.00	1.17 (0.94–1.46)
*P* value		0.0319			0.1670			0.2905			0.1708
**Down syndrome**											
*n* (/10000)	6040 (5.49)	620 (5.49)		6170 (5.46)	490 (5.79)		6243 (5.47)	416 (5.75)		6232 (5.45)	398 (5.79)
RR (95% CI)	1.00	1.03 (0.95–1.13)		1.00	1.03 (0.93–1.14)		1.00	0.99 (0.89–1.10)		1.00	1.00 (0.90–1.12)
*P* value		0.4795			0.5453			0.8583			0.9346
**Suspected chromosomal disorder**											
*n* (/10000)	4814 (4.37)	597 (5.28)		4940 (4.37)	469 (5.54)		4999 (4.38)	410 (5.66)		4964 (4.34)	380 (5.53)
RR (95% CI)	1.00	1.09 (0.99–1.19)		1.00	1.09 (0.98–1.20)		1.00	1.09 (0.98–1.21)		1.00	1.08 (0.97–1.21)
*P* value		0.0681			0.1040			0.1109			0.1595
**Anencephaly**											
*n* (/10000)	978 (0.89)	143 (1.27)		1010 (0.89)	111 (1.31)		1022 (0.90)	99 (1.37)		990 (0.87)	99 (1.44)
RR (95% CI)	1.00	1.02 (0.85–1.24)		1.00	0.98 (0.79–1.21)		1.00	1.00 (0.80–1.25)		1.00	1.14 (0.91–1.42)
*P* value		0.8090			0.8363			0.9929			0.2429
**Cyanotic congenital heart disease**											
*n* (/10000)	5904 (5.36)	719 (6.36)		6075 (5.38)	551 (6.51)		6172 (5.41)	453 (6.26)		6150 (5.38)	428 (6.23)
RR (95% CI)	1.00	0.97 (0.89–1.05)		1.00	0.94 (0.86–1.03)		1.00	0.88 (0.79–0.97)		1.00	0.88 (0.80–0.98)
*P* value		0.4612			0.2049			0.0111			0.0184
**Meningomyelocele/Spina bifida**											
*n* (/10000)	1555 (1.41)	183 (1.62)		1589 (1.41)	150 (1.77)		1611 (1.41)	128 (1.77)		1596 (1.40)	120 (1.75)
RR (95% CI)	1.00	0.89 (0.76–1.05)		1.00	0.95 (0.80–1.14)		1.00	0.93 (0.77–1.13)		1.00	0.94 (0.78–1.15)
*P* value		0.1726			0.5794			0.4770			0.5681
**Any outcome**^**⁑**^											
*n* (/10000)	18837 (17.11)	3208 (28.39)		19562 (17.32)	2482 (29.33)		12981 (11.37)	1582 (21.85)		13022 (11.39)	1485 (21.61)
RR (95% CI)	1.00	1.30 (1.25–1.36)		1.00	1.30 (1.24–1.36)		1.00	1.35 (1.27–1.43)		1.00	1.34 (1.26–1.42)
*P* value		< 0.0001			< 0.0001			< 0.0001			< 0.0001

Poisson regression models were adjusted for maternal age, race/ethnicity, educational level, marital status, maternal pre-pregnancy body mass index, eclampsia, gestational hypertension and diabetes, parity, infant sex, gestational age at delivery, and total number of prenatal care visits

*Only for boys

^⁑^Any outcome was defined as having any one of birth congenital anomalies that were significantly associated with maternal cigarette smoking

*CI* confidence interval, *RR* risk ratio

Regarding overall congenital anomalies (defined as having any one of the congenital malformations above significantly associated with maternal cigarette smoking), the adjusted RRs (95% CIs) were 1.30 (1.25–1.36) for women who smoked before pregnancy and 1.30 (1.24–1.36), 1.35 (1.27–1.43), and 1.34 (1.26–1.42) for women who smoked in the first, second, and third trimesters, respectively (all *p* < 0.0001).

Considering smoking cessation during pregnancy, we found that compared with women who kept smoking before and throughout pregnancy, maternal smokers who quitted smoking in the first, second, or third trimester did not have a significantly reduced risk of overall congenital anomalies, with adjusted RRs (95% CI) of 0.99(0.91–1.08), 1.06(0.95–1.19), and 0.93 (0.78–1.10), respectively (Additional file 1: Table S1). In contrast, those who never smoked before and during pregnancy had a significantly lower risk of overall birth congenital anomalies (RR 0.77, 95% CI 0.73–0.81).

### Associations between the intensity of maternal cigarette smoking and birth congenital anomalies

Compared with non-smokers, infants born to women who consumed any number of cigarettes per day before or during pregnancy, even only 1–5 cigarettes per day, had an increased risk of four subtypes of congenital anomalies, including gastroschisis, limb reduction defect, cleft lip with or without cleft palate, and cleft palate alone. For example, the adjusted RRs (95% CIs) of cleft lip with or without cleft palate of infants born to women who smoked 1–5, 6–10, and ≥ 11 cigarettes per day before pregnancy were 1.33 (1.16–1.52), 1.19 (1.05–1.35), and 1.32 (1.18–1.47), respectively. The corresponding values were 1.26 (1.10–1.45), 1.21 (1.07–1.38), and 1.33 (1.15–1.53) for women who smoked in the first trimester; 1.22 (1.06–1.41), 1.26 (1.10–1.43), and 1.33 (1.11–1.59) for women who smoked in the second trimester; and 1.20 (1.05–1.38), 1.23 (1.07–1.41), and 1.33 (1.10–1.63) for women who smoked in the third trimester. For congenital diaphragmatic hernia and hypospadias, the significant association was observed for the specific intensity of maternal cigarette smoking in specific trimester (Tables 3, 4, 5, and 6). The statistical significance still existed in most groups even after the Bonferroni correction (the corrected *p* values ranged from 0.0024 (0.05/(3*7)) to 0.0028 (0.05/(3*6)) when subgroups ranged from 21 (Tables 3 and 4) to 18 (Tables 5 and 6), respectively).

**Table 3:** Associations of the intensity of maternal cigarette smoking before pregnancy with birth congenital anomalies

	Number of cigarettes consumed per day			
	0	1–5	6–10	≥11
**Congenital diaphragmatic hernia**				
*n* (/10000)	1263( 1.15)	36 (1.23)	76 (1.98)	93 (2.05)
RR (95% CI)	1.00	0.99 (0.70–1.38)	1.47 (1.16–1.87)	1.47 (1.18–1.84)
*P* value		0.9311	0.0016	0.0006
**Gastroschisis**				
*n* (/10000)	2116 (1.92)	157 (5.37)	203 (5.29)	244 (5.37)
RR (95% CI)	1.00	1.67 (1.42–1.98)	1.53 (1.31–1.78)	1.47 (1.28–1.69)
*P* value		< 0.0001	< 0.0001	< 0.0001
**Limb reduction defect**				
*n* (/10000)	1384 (1.26)	61 (2.09)	92 (2.40)	119 (2.62)
RR (95% CI)	1.00	1.39 (1.07–1.81)	1.51 (1.21–1.88)	1.60 (1.31–1.95)
*P* value		0.0131	0.0002	< 0.0001
**Cleft lip with or without cleft palate**				
*n* (/10000)	5462 (4.96)	219 (7.50)	283 (7.37)	382 (8.41)
RR (95% CI)	1.00	1.33 (1.16–1.52)	1.19 (1.05–1.35)	1.32 (1.18–1.47)
*P* value		< 0.0001	0.0053	< 0.0001
**Cleft palate alone**				
*n* (/10000)	2479 (2.25)	102 (3.49)	162 (4.22)	225 (4.96)
RR (95% CI)	1.00	1.31 (1.07–1.60)	1.40 (1.19–1.65)	1.58 (1.37–1.82)
*P* value		0.0095	< 0.0001	< 0.0001
Hypospadias*				
*n* (/10000)	6680 (11.86)	223 (14.92)	270 (13.75)	382 (16.48)
RR (95% CI)	1.00	1.20 (1.05–1.38)	1.04 (0.91–1.17)	1.21 (1.09–1.35)
*P* value		0.0070	0.5885	0.0005
**Any outcome**^**⁑**^				
*n* (/10000)	18837 (17.11)	769 (26.32)	1048 (27.31)	1391 (30.64)
RR (95% CI)	1.00	1.31 (1.22–1.41)	1.25 (1.17–1.33)	1.35 (1.28–1.43)
*P* value		< 0.0001	< 0.0001	< 0.0001

Poisson regression models were adjusted for maternal age, race/ethnicity, educational level, marital status, maternal pre-pregnancy body mass index, eclampsia, gestational hypertension and diabetes, parity, infant sex, gestational age at delivery, and total number of prenatal care visits

*Only for boys

^⁑^Any outcome was defined as having any one of congenital diaphragmatic hernia, gastroschisis, limb reduction defect, cleft lip with or without cleft palate, cleft palate alone, and hypospadias

*CI* confidence interval, *RR* risk ratio

**Table 4:** Associations of the intensity of maternal cigarette smoking in the first trimester with birth congenital anomalies

	Number of cigarettes consumed per day			
	0	1–5	6–10	≥ 11
**Congenital diaphragmatic hernia**				
*n* (/10000)	1314 (1.16)	39 (1.41)	69 (2.05)	46 (1.97)
RR (95% CI)	1.00	1.06 (0.76–1.46)	1.42 (1.10–1.83)	1.33 (0.98–1.80)
*P* value		0.7408	0.0063	0.0647
**Gastroschisis**				
*n* (/10000)	2263 (2.00)	149 (5.40)	179 (5.31)	129 (5.53)
RR (95% CI)	1.00	1.48 (1.25–1.75)	1.39 (1.18–1.63)	1.46 (1.22–1.76)
*P* value		< 0.0001	< 0.0001	< 0.0001
**Limb reduction defect**				
*n* (/10000)	1433 (1.27)	59 (2.14)	92 (2.73)	71 (3.04)
RR (95% CI)	1.00	1.35 (1.03–1.76)	1.63 (1.31–2.03)	1.77 (1.38–2.27)
*P* value		0.0287	< 0.0001	< 0.0001
**Cleft lip with or without cleft palate**				
*n* (/10000)	5660 (5.01)	208 (7.54)	268 (7.95)	209 (8.96)
RR (95% CI)	1.00	1.26 (1.10–1.45)	1.21 (1.07–1.38)	1.33 (1.15–1.53)
*P* value		0.0012	0.0028	0.0001
**Cleft palate alone**				
*n* (/10000)	2554 (2.26)	108 (3.91)	175 (5.19)	130 (5.58)
RR (95% CI)	1.00	1.39 (1.14–1.69)	1.66 (1.41–1.95)	1.73 (1.44–2.08)
*P* value		0.0010	< 0.0001	< 0.0001
Hypospadias*				
*n* (/10000)	6910 (11.96)	179 (12.68)	285 (16.51)	182 (15.23)
RR (95% CI)	1.00	0.99 (0.85–1.16)	1.23 (1.09–1.39)	1.12 (0.96–1.30)
*P* value		0.9344	0.0011	0.1489
**Any outcome**^**⁑**^				
*n* (/10000)	19562 (17.32)	726 (26.31)	1029 (30.52)	727 (31.18)
RR (95% CI)	1.00	1.23 (1.14–1.33)	1.33 (1.24–1.42)	1.33 (1.23–1.43)
*P* value		< 0.0001	< 0.0001	< 0.0001

Poisson regression models were adjusted for maternal age, race/ethnicity, educational level, marital status, maternal pre-pregnancy body mass index, eclampsia, gestational hypertension and diabetes, parity, infant sex, gestational age at delivery, and total number of prenatal care visits

*Only for boys

^⁑^Any outcome was defined as having any one of congenital diaphragmatic hernia, gastroschisis, limb reduction defect, cleft lip with or without cleft palate, cleft palate alone, and hypospadias

*CI* confidence interval, *RR* risk ratio

**Table 5:** Associations of the intensity of maternal cigarette smoking in the second trimester with birth congenital anomalies

	Number of cigarettes consumed per day			
	0	1–5	6–10	≥11
**Congenital diaphragmatic hernia**				
*n* (/10000)	1327 (1.16)	49 (1.79)	58 (1.88)	33 (2.35)
RR (95% CI)	1.00	1.32 (0.98–1.76)	1.29 (0.98–1.70)	1.56 (1.09–2.22)
*P* value		0.0661	0.0660	0.0144
**Gastroschisis**				
*n* (/10000)	2332 (2.04)	156 (5.69)	163 (5.27)	69 (4.91)
RR (95% CI)	1.00	1.50 (1.27–1.77)	1.37 (1.16–1.61)	1.30 (1.02–1.67)
*P* value		< 0.0001	0.0002	0.0335
**Limb reduction defect**				
*n* (/10000)	1468 (1.29)	60 (2.19)	84 (2.72)	43 (3.06)
RR (95% CI)	1.00	1.32 (1.01–1.71)	1.56 (1.24–1.96)	1.69 (1.24–2.31)
*P* value		0.0419	0.0002	0.0010
**Cleft lip with or without cleft palate**				
*n* (/10000)	5747 (5.03)	206 (7.51)	261 (8.44)	130 (9.25)
RR (95% CI)	1.00	1.22 (1.06–1.41)	1.26 (1.10–1.43)	1.33 (1.11–1.59)
*P* value		0.0062	0.0005	0.0016
**Cleft palate alone**				
*n* (/10000)	2624 (2.3)	105 (3.83)	166 (5.37)	71 (5.05)
RR (95% CI)	1.00	1.28 (1.05–1.57)	1.63 (1.38–1.92)	1.50 (1.18–1.91)
*P* value		0.0143	< 0.0001	0.0011
Any outcome*				
*n* (/10000)	12981 (11.37)	558 (20.34)	696 (22.51)	328 (23.35)
RR (95% CI)	1.00	1.32 (1.21–1.44)	1.36 (1.26–1.47)	1.38 (1.23–1.54)
*P* value		< 0.0001	< 0.0001	< 0.0001

Poisson regression models were adjusted for maternal age, race/ethnicity, educational level, marital status, maternal pre-pregnancy body mass index, eclampsia, gestational hypertension and diabetes, parity, infant sex, gestational age at delivery, and total number of prenatal care visits

*Any outcome was defined as having any one of congenital diaphragmatic hernia, gastroschisis, limb reduction defect, cleft lip with or without cleft palate, and cleft palate alone

*CI* confidence interval, *RR* risk ratio

**Table 6:** Associations of the intensity of maternal cigarette smoking in the third trimester with birth congenital anomalies

	Number of cigarettes consumed per day			
	0	1–5	6–10	≥ 11
**Congenital diaphragmatic hernia**				
*n* (/10000)	1325 (1.16)	52 (1.70)	54 (2.02)	24 (2.08)
RR (95% CI)	1.00	1.27 (0.95–1.68)	1.39 (1.05–1.84)	1.37 (0.91–2.07)
*P* value		0.1031	0.0228	0.1308
**Gastroschisis**				
*n* (/10000)	2350 (2.06)	175 (5.73)	132 (4.95)	51 (4.43)
RR (95% CI)	1.00	1.59 (1.35–1.87)	1.32 (1.10–1.59)	1.20 (0.91–1.59)
*P* value		<0.0001	0.0025	0.2043
**Limb reduction defect**				
*n* (/10000)	1474 (1.29)	70 (2.29)	73 (2.74)	34 (2.95)
RR (95% CI)	1.00	1.38 (1.08–1.77)	1.55 (1.21–1.98)	1.61 (1.14–2.28)
*P* value		0.0100	0.0004	0.0075
**Cleft lip with or without cleft palate**				
*n* (/10000)	5762 (5.04)	229 (7.50)	223 (8.36)	108 (9.38)
RR (95% CI)	1.00	1.20 (1.05–1.38)	1.23 (1.07–1.41)	1.33 (1.10–1.63)
*P* value		0.0075	0.0032	0.0033
**Cleft palate alone**				
*n* (/10000)	2630 (2.3)	121 (3.96)	150 (5.62)	59 (5.12)
RR (95% CI)	1.00	1.32 (1.09–1.59)	1.70 (1.43–2.02)	1.52 (1.16–1.97)
*P* value		0.0042	<0.0001	0.0020
Any outcome*				
*n* (/10000)	13022 (11.39)	624 (20.45)	597 (22.38)	264 (22.92)
RR (95% CI)	1.00	1.33 (1.22–1.44)	1.35 (1.24–1.47)	1.35 (1.19–1.52)
*P* value		< 0.0001	< 0.0001	< 0.0001

Poisson regression models were adjusted for maternal age, race/ethnicity, educational level, marital status, maternal pre-pregnancy body mass index, eclampsia, gestational hypertension and diabetes, parity, infant sex, gestational age at delivery, and total number of prenatal care visits

*Any outcome was defined as having any one of congenital diaphragmatic hernia, gastroschisis, limb reduction defect, cleft lip with or without cleft palate, and cleft palate alone

*CI* confidence interval, *RR* risk ratio

Regarding overall congenital anomalies, the adjusted RRs (95% CIs) for women who smoked 1–5, 6–10, and ≥ 11 cigarettes per day before pregnancy were 1.31 (1.22–1.41), 1.25 (1.17–1.33), and 1.35 (1.28–1.43), respectively. The corresponding values were 1.23 (1.14–1.33), 1.33 (1.24–1.42), and 1.33 (1.23–1.43) for women who smoked cigarettes in the first trimester; 1.32 (1.21–1.44), 1.36 (1.26–1.47), and 1.38 (1.23–1.54) for women who smoked in the second trimester; and 1.33 (1.22–1.44), 1.35 (1.24–1.47), and 1.35 (1.19–1.52) for women who smoked in the third trimester (all *p* < 0.0001) (Tables 3, 4, 5, and 6).

### Sensitivity analyses

Sensitivity analyses yielded similar results to our main analyses, including by excluding women with cesarean section or excluding women with eclampsia, gestational hypertension or diabetes, excluding women with preterm delivery, and based on imputation analyses. All these analyses above showed that maternal cigarette smoking before pregnancy or during each trimester significantly increased the risk of six subtypes of congenital anomalies mentioned before (Additional file 1: Table S2).

## Discussion

In this nationwide population-based cohort study of more than 12 million live births in the USA, we found that maternal cigarette smoking, even at a low intensity (1–5 cigarettes per day), before pregnancy or during each trimester of pregnancy significantly increased the risk of six specific subtypes of congenital anomalies (i.e., congenital diaphragmatic hernia, gastroschisis, limb reduction defect, cleft lip with or without cleft palate, cleft palate alone, and hypospadias). Compared with persistent smokers before and throughout pregnancy, a significantly lower risk of congenital anomalies was observed for never smokers, but no reduced risk was observed for women who smoked before pregnancy and quitted during each trimester of pregnancy.

### Comparisons with other studies

In the present study, we found an increased risk of overall congenital anomalies in offspring born to mothers who smoked cigarettes during pregnancy, which contrasts with a previous large population-based cohort study using data from the Danish Medical Birth Register, which did not find an overall increased risk of congenital anomalies (OR 0.98, 95% CI 0.95–1.01) [13]. It should be noted that the Danish study included a smaller sample size (*n* = 838,265) than ours (*n* = 12,144,972, especially for rare outcomes, for example, the risk estimate for omphalocele in the Danish study was based on 25 exposed cases). The Danish study also did not adjust for potentially important covariates which we have adjusted for in our analysis (e.g., race/ethnicity, maternal pre-pregnancy BMI, infant sex, eclampsia, gestational hypertension, and gestational diabetes). In addition, disparities in definitions of “overall congenital anomalies” may also contribute to the inconsistent results. However, when we used having any of the 12 congenital anomalies to define “overall congenital anomalies,” we still found an increased risk of overall congenital anomalies for maternal smoking (data not shown).

Since different anomalies can have different etiologies, defect-specific analyses may better reflect the association between maternal cigarette smoking and congenital anomalies. A meta-analysis of 6 case-control studies (4209 malformed cases and 10,646 controls) published between 2000 and 2010 revealed that maternal smoking during pregnancy significantly increased the risk of cleft lip with or without cleft palate (OR 1.48, 95% CI 1.36–1.61) [25]. The Danish study [13] mentioned above also showed that maternal smoking in pregnancy was associated with an increased risk of this anomaly (OR 1.36, 95% CI 1.18–1.56), similar to our results. We also observed an increased risk of limb reduction defects, congenital diaphragmatic hernia, and gastroschisis among women who smoked cigarettes, consistent with a meta-analysis by Hackshaw et al. [26] which identified 172 studies (173,687 malformed cases and 11,674,332 controls; conducted between 1959 and 2010; limb reduction defects: OR 1.26, 95% CI 1.15–1.39; hernia: OR 1.40, 95% CI 1.23–1.59; gastroschisis: OR 1.50, 95% CI 1.28–1.76). However, the Danish study [13] reported a marginally reduced risk of gastroschisis (OR 0.89, 95% CI 0.56–1.39; based on only 25 exposed cases) and no association with diaphragmatic hernia (OR 1.00, 95% CI 0.68–1.49; based on only 32 exposed cases). In addition, the meta-analysis by Hackshaw et al. [26] reported a reduced risk for hypospadias (OR 0.90, 95% CI 0.85–0.95), similar with the Danish study [13] which reported a marginally reduced risk of hypospadias (OR 0.94, 95% CI 0.83–1.06), inconsistent with our results (showing a significantly increased risk). Due to the large number of cases in our study, we do not believe the observed increased risk of hypospadias was entirely due to chance although the association needs to be warranted in further studies. Furthermore, in contrast to previous studies [13, 27], we did not detect a significant association between maternal cigarette smoking during pregnancy and cyanotic congenital heart disease. This result may be expected, as congenital heart defects consist of many heterogeneous subtypes that have different genetic and embryological origins. Further research should examine the effects of maternal smoking on specific subtypes of congenital heart defects.

In addition to clarifying the associations of maternal cigarette smoking with birth congenital anomalies, a novel finding of this study was that maternal cigarette smoking either before pregnancy or during each trimester of pregnancy significantly increased the risk of birth congenital anomalies, suggesting that there is no safe period of maternal smoking, which has been seldom reported in the previous studies mentioned above. It is interesting that the associations of smoking with congenital anomalies did not change significantly according to different trimesters. One speculative, possible explanation would be that the detrimental effects of maternal smoking on congenital anomalies mainly originate from an earlier period in pregnancy: morphogenesis is influenced mainly during the first days of embryogenesis, whereas later days of pregnancy involve mostly about growth of the embryo. The observed similar association between maternal smoking in the second and third trimesters of pregnancy and congenital anomalies might be due to the fact that the majority of women who smoked in early pregnancy continued to smoke cigarettes throughout pregnancy.

Another important finding of this study was that smoking exposure within three months before pregnancy, but with cessation during the pregnancy, had the similar risk of birth congenital anomalies compared with persistent smokers before and during pregnancy. One possible explanation was the detrimental action of maternal smoking on some genes—genetic or epigenetic—of the ovules (i.e., the potentially critical periods for preventing congenital anomalies seemed to be pre-conception and/or during the first days of pregnancy) [28]. Another speculative explanation was the known longer half-life of nicotine [29] and the nicotine withdrawal effect [30]. In contrast, women who never smoked before and during pregnancy were less likely to have birth congenital anomalies compared to persistent smokers throughout pregnancy. We believe that these findings have important public health implications. Many women who smoke cigarettes believe that it is acceptable to smoke before pregnancy or in the first trimester [31]. Besides, when a woman finds herself pregnant and begins prenatal care, many fetal organs may have already been formed, and smoking cessation might be too late to prevent congenital anomalies. Therefore, there is an urgent need to strengthen pre-pregnancy health services to help minimize the risk of congenital anomalies.

The third important finding of our study was that mothers who smoked only few cigarettes (1–5 cigarettes per day) had an increased risk of congenital anomalies compared with non-smokers, suggesting there is no safe level of cigarette smoking for pregnant women. Similarly, Chung, et al. [18] used the 1996 US Natality database (2207 cases and 4414 controls) found an increased risk for cleft lip with or without cleft palate among light smokers (1–10 cigarettes per day; OR 1.50, 95% CI 1.28–1.76). Another large study used Swedish register data (*n* = 1,413,811; 1983–1996) showed a marginally positive association between light smokers (< 10 cigarettes per day) and any malformation (OR 1.02, 95% CI 0.98–1.05) [32]. However, the two studies used smoking 1–9 cigarettes per day to define light smoking. The Danish study [13] used smoking 1–5 cigarettes per day to define light smoking and showed that infants born to mothers who smoked a low dose of cigarettes per day were at a marginally reduced risk of major malformations (OR 0.96, 95% CI 0.92–1.01). These previous studies also did not distinguish the effect of intensity of maternal smoking in different periods (before or during different trimesters of pregnancy) on congenital anomalies, while our study showed the similar effect highlighting maternal smoking even at a low intensity during any period increased the risk of congenital anomalies. It should be noted that our study did not find clear dose-response associations between maternal smoking before or during the pregnancy and birth congenital anomalies, similar to a previous study on maternal smoking and congenital heart defects by Alverson et al. (2525 malformations and 3435 controls) [14]. Another older study showed no dose-response association between maternal smoking and cleft lip with or without cleft palate [33]. The possible explanations included the competing risks (e.g., abortion and stillbirth) caused by high doses of consumed cigarettes [34], which might bias the relationship with congenital anomalies toward null. In addition, other unmeasured confounding factors might have also biased our findings.

Finally, it should be mentioned that magnitudes of the positive association between maternal smoking and congenital anomalies in our study were somewhat higher than those in previous studies mentioned above. We speculate that this may be due to variability in exposure assessment (i.e., cigarette smoking) and case ascertainment (i.e., congenital anomalies). In addition, many previous studies were based on relatively small number of cases on congenital anomalies and with adjustment for a limited number of potential confounders. Further large well-designed prospective cohort studies are needed to warrant the observed association and to examine the possible explanations.

### Strengths and limitations

The main strengths of this study include the population-based cohort design, the large sample size of over 12 million live births, the detailed examination of the associations between the timing and intensity of maternal cigarette smoking and many congenital anomalies subtypes considered (including rare subtypes), the adjustment for several (but not all possible) covariates, and the sensitivity analyses used to confirm our findings. However, this study also has several limitations. First, there were limitations to use birth certificate data for assessing the association with birth defects. Data on pregnancy complications, and comorbid conditions tended to be underreported on the birth certificates [35]. However, a previous study used data from the West Virginia (1990–2009) to assess the effects of misclassification bias on reported congenital anomalies based on birth certificate data, and the results showed that specified birth defects collected using checked-box format with definite criteria on the 2003 version birth certificate showed consistent patterns over time [36]. In addition, several other studies have been performed to assess the validity of birth certificate data for effect estimation [22, 37] and the results suggest that birth certificate data may be useful for exploratory studies assessing the association between birth congenital anomalies and some risk factors. Second, maternal cigarette smoking was self-reported and recall bias might have occurred. However, a previous study showed that self-reported number of cigarettes were highly correlated with objectively measured cotinine during pregnancy [38]. Moreover, pregnant women were unwilling to report smoking if they had illness conditions around the time of delivery in the hospital. This also could cause some bias or misclassification. Third, information on non-daily cigarette smoking (< 1 cigarette per day) before or during pregnancy was unavailable on the birth certificate. Fourth, for “maternal pre-pregnancy smoking,” the NVSS only collected information on smoking within 3 months before pregnancy. Thus, we could not differentiate women who never smoked from those who did within 3 months before pregnancy. We then also could not compare the risk of congenital anomalies for smoking cessation before pregnancy vs. persist smoking, and the former period is the potentially critical period for preventing congenital anomalies. Fifth, the NVSS only collected data on congenital anomalies identified at birth. However, some types of congenital anomalies could not be identified at that time, and usually appear in childhood or even adulthood. This may cause outcome misclassification (i.e., the infants who did not have congenital anomalies at birth but diagnosed in later life were treated as not having congenital anomalies in this study). Furthermore, we only included live births and did not account for congenital anomalies in aborted fetuses and stillbirths, which may lead to selection bias (i.e., newborns with malformations among maternal smokers may be underrepresented in a study of live births). Finally, although we have adjusted for a range of potential confounders, we could not completely rule out the possibility of residual confounding or confounding from unmeasured confounders. For example, one important potential confounder that we did not adjust for was second-hand smoke exposure, which has been shown to be nearly as deleterious as active smoking to the developing fetus [39]. Another important potential confounder was alcohol consumption as drinking was associated with both smoking and embryo malformations [40]. In addition, we only adjusted for maternal race/ethnicity without considering that of the infant. It is possible that the infant may have a different racial make-up than the mother, especially if one race/ethnicity was more susceptible to the effects of smoking but only race/ethnicity of mother is considered, this could cause some bias or misclassification.

## Conclusions

In conclusion, our findings showed that maternal cigarette smoking, even at a low intensity (1–5 cigarettes per day), before pregnancy or during either trimester of pregnancy significantly increased the risk of birth congenital anomalies. Women who smoked before pregnancy and quitted during each trimester of pregnancy still had an increased risk of congenital anomalies in offspring than women who did not smoke before and throughout pregnancy. Therefore, smoking cessation interventions should be implemented before pregnancy. Early initiation of pre-pregnancy counseling is of paramount importance which should include an explanation of the adverse effects of cigarette smoking on both mothers and their children including congenital anomalies and suggest on not to start smoking (for never smokers) or to stop smoking completely (for current smokers) both before and during pregnancy. The current study also provided strong evidence to strengthen the recommendation proposed by the US public health service (i.e., 5A’s: ask, advise, assess, assist, and arrange) for health care providers to help maternal smokers to quit smoking [41]. Interventions should also emphasize the detrimental effects of even light smoking for pregnant women.

## Supplementary Information

**Additional file 1.** Supplementary results. **Table S1.** Associations of maternal smoking cessation during pregnancy with overall birth congenital anomalies. **Table S2.** Sensitivity analyses of associations between the timing of maternal cigarette smoking and birth congenital anomalies.

## Data Availability

The birth certificate data from the National Vital Statistics System are available at the official website (https:// www.cdc.gov/nchs/nvss/births.htm).

## Abbreviations

BMI: Body mass index
CI: Confidence interval
NVSS: National Vital Statistics System
OR: Odds ratios
RR: Risk ratios
