# Supplementary material for: Maternal cigarette smoking before or during pregnancy increases the risk of birth congenital anomalies: a population-based retrospective cohort study of 12 million mother-infant pairs
Source: BMC Med. 2022 Jan 11;20:4. doi: 10.1186/s12916-021-02196-x (PMC8750764; doi:10.1186/s12916-021-02196-x)
Supplement: Supplementary file 1 — Additional file 1. Supplementary results. Table S1. Associations of maternal smoking cessation during pregnancy with overall birth congenital anomalies. Table S2. Sensitivity analyses of associations between the timing of maternal cigarette smoking and birth congenital anomalies. [file 12916_2021_2196_MOESM1_ESM.doc]

| **Table S1. Associations of maternal smoking cessation during pregnancy with overall birth congenital anomalies**^*^ | | | |
| --- | --- | --- | --- |
| **Group** | **N** | RR (95% CI) | *P* value |
| Persistent smoker | 661111 | 1.00 |  |
| Never smoker | 10976945 | 0.77(0.73-0.81) | <0.0001 |
| Quit in the first trimester | 277598 | 0.99(0.91-1.08) | 0.7946 |
| Quit in the second trimester | 120559 | 1.06(0.95-1.19) | 0.3048 |
| Quit in the third trimester | 47459 | 0.93(0.78-1.10) | 0.3985 |
| Poisson regression models were adjusted for maternal age, race/ethnicity, educational level, marital status, maternal pre-pregnancy body mass index, eclampsia, gestational hypertension and diabetes, parity, infant sex, gestational age at delivery, and total number of prenatal care visits; | | | |
| ^*^ Overall birth congenital anomalies was defined as having any one of congenital diaphragmatic hernia, gastroschisis, limb reduction defect, cleft lip with or without cleft palate, cleft palate alone, and hypospadias. | | | |

| **Table S2. Sensitivity analyses of associations between the timing of maternal cigarette smoking and birth congenital anomalies** | | | | | | | | | | | |
| --- | --- | --- | --- | --- | --- | --- | --- | --- | --- | --- | --- |
| **Outcomes** | Smoking before pregnancy | |  | Smoking in the first trimester | |  | Smoking in the second trimester | |  | Smoking in the third trimester | |
|  | No | Yes |  | No | Yes |  | No | Yes |  | No | Yes |
| ***Exclusion of women with caesarean section***^*^ |  |  |  |  |  |  |  |  |  |  |  |
| Congenital diaphragmatic hernia | 1.00 | 1.38(1.12-1.70) |  | 1.00 | 1.33(1.05-1.68) |  | 1.00 | 1.39(1.09-1.77) |  | 1.00 | 1.37(1.07-1.76) |
| Gastroschisis | 1.00 | 1.67(1.46-1.91) |  | 1.00 | 1.54(1.33-1.79) |  | 1.00 | 1.54(1.32-1.80) |  | 1.00 | 1.52(1.30-1.79) |
| Limb reduction defect | 1.00 | 1.38(1.14-1.67) |  | 1.00 | 1.45(1.18-1.79) |  | 1.00 | 1.31(1.05-1.64) |  | 1.00 | 1.28(1.02-1.61) |
| Cleft lip with or without cleft palate | 1.00 | 1.32(1.20-1.45) |  | 1.00 | 1.29(1.16-1.44) |  | 1.00 | 1.29(1.16-1.44) |  | 1.00 | 1.24(1.10-1.39) |
| Cleft palate alone | 1.00 | 1.46(1.28-1.67) |  | 1.00 | 1.64(1.42-1.89) |  | 1.00 | 1.49(1.28-1.74) |  | 1.00 | 1.51(1.29-1.76) |
| Hypospadias**^†^** | 1.00 | 1.15(1.05-1.27) |  | 1.00 | 1.15(1.03-1.29) |  | ― | ― |  | ― | ― |
| Any outcome^⁑^ | 1.00 | 1.32(1.26-1.39) |  | 1.00 | 1.32(1.25-1.40) |  | 1.00 | 1.37(1.28-1.47) |  | 1.00 | 1.34(1.24-1.44) |
|  |  |  |  |  |  |  |  |  |  |  |  |
| ***Exclusion of women with eclampsia, gestational hypertension or diabetes*^§^** |  |  |  |  |  |  |  |  |  |  |  |
| Congenital diaphragmatic hernia | 1.00 | 1.36(1.15-1.61) |  | 1.00 | 1.28(1.06-1.55) |  | 1.00 | 1.35(1.11-1.65) |  | 1.00 | 1.33(1.08-1.64) |
| Gastroschisis | 1.00 | 1.55(1.40-1.72) |  | 1.00 | 1.43(1.28-1.61) |  | 1.00 | 1.42(1.26-1.60) |  | 1.00 | 1.44(1.27-1.63) |
| Limb reduction defect | 1.00 | 1.43(1.23-1.67) |  | 1.00 | 1.48(1.26-1.75) |  | 1.00 | 1.42(1.19-1.70) |  | 1.00 | 1.40(1.17-1.68) |
| Cleft lip with or without cleft palate | 1.00 | 1.28(1.18-1.40) |  | 1.00 | 1.27(1.16-1.40) |  | 1.00 | 1.26(1.15-1.39) |  | 1.00 | 1.24(1.12-1.37) |
| Cleft palate alone | 1.00 | 1.48(1.32-1.66) |  | 1.00 | 1.60(1.42-1.81) |  | 1.00 | 1.46(1.28-1.67) |  | 1.00 | 1.49(1.30-1.70) |
| Hypospadias**^†^** | 1.00 | 1.14(1.05-1.24) |  | 1.00 | 1.14(1.04-1.25) |  | ― | ― |  | ― | ― |
| Any outcome^⁑^ | 1.00 | 1.30(1.25-1.36) |  | 1.00 | 1.30(1.24-1.36) |  | 1.00 | 1.34(1.26-1.42) |  | 1.00 | 1.32(1.25-1.41) |
|  |  |  |  |  |  |  |  |  |  |  |  |
| ***Exclusion of infants with preterm birth***^*^ |  |  |  |  |  |  |  |  |  |  |  |
| Congenital diaphragmatic hernia | 1.00 | 1.21(1.00-1.47) |  | 1.00 | 1.11(0.89-1.39) |  | 1.00 | 1.15(0.91-1.44) |  | 1.00 | 1.11(0.87-1.41) |
| Gastroschisis | 1.00 | 1.38(1.17-1.62) |  | 1.00 | 1.25(1.04-1.50) |  | 1.00 | 1.15(0.95-1.40) |  | 1.00 | 1.12(0.92-1.37) |
| Limb reduction defect | 1.00 | 1.50(1.27-1.77) |  | 1.00 | 1.62(1.36-1.94) |  | 1.00 | 1.47(1.21-1.78) |  | 1.00 | 1.46(1.20-1.78) |
| Cleft lip with or without cleft palate | 1.00 | 1.29(1.19-1.40) |  | 1.00 | 1.24(1.13-1.37) |  | 1.00 | 1.24(1.12-1.37) |  | 1.00 | 1.22(1.10-1.35) |
| Cleft palate alone | 1.00 | 1.47(1.31-1.65) |  | 1.00 | 1.61(1.42-1.83) |  | 1.00 | 1.54(1.34-1.76) |  | 1.00 | 1.56(1.36-1.78) |
| Hypospadias**^†^** | 1.00 | 1.17(1.08-1.27) |  | 1.00 | 1.14(1.04-1.26) |  | ― | ― |  | ― | ― |
| Any outcome^⁑^ | 1.00 | 1.28(1.22-1.34) |  | 1.00 | 1.27(1.21-1.34) |  | 1.00 | 1.30(1.21-1.39) |  | 1.00 | 1.28(1.20-1.38) |
|  |  |  |  |  |  |  |  |  |  |  |  |
| ***Analysis after imputation***^#*^ |  |  |  |  |  |  |  |  |  |  |  |
| Congenital diaphragmatic hernia | 1.00 | 1.28(1.10-1.48) |  | 1.00 | 1.25(1.06-1.47) |  | 1.00 | 1.27(1.07-1.51) |  | 1.00 | 1.27(1.07-1.52) |
| Gastroschisis | 1.00 | 1.56(1.42-1.71) |  | 1.00 | 1.47(1.33-1.63) |  | 1.00 | 1.43(1.29-1.60) |  | 1.00 | 1.44(1.29-1.61) |
| Limb reduction defect | 1.00 | 1.52(1.32-1.74) |  | 1.00 | 1.57(1.35-1.82) |  | 1.00 | 1.49(1.27-1.74) |  | 1.00 | 1.46(1.24-1.72) |
| Cleft lip with or without cleft palate | 1.00 | 1.28(1.19-1.38) |  | 1.00 | 1.26(1.17-1.37) |  | 1.00 | 1.26(1.16-1.38) |  | 1.00 | 1.25(1.15-1.37) |
| Cleft palate alone | 1.00 | 1.45(1.31-1.61) |  | 1.00 | 1.56(1.40-1.75) |  | 1.00 | 1.45(1.29-1.63) |  | 1.00 | 1.48(1.32-1.67) |
| Hypospadias**^†^** | 1.00 | 1.19(1.11-1.28) |  | 1.00 | 1.15(1.06-1.25) |  | ― | ― |  | ― | ― |
| Any outcome^⁑^ | 1.00 | 1.32(1.27-1.37) |  | 1.00 | 1.30(1.25-1.36) |  | 1.00 | 1.34(1.27-1.41) |  | 1.00 | 1.34(1.27-1.41) |
| ^*^Poisson regression models were adjusted for maternal age, race/ethnicity, educational level, marital status, maternal pre-pregnancy body mass index, eclampsia, gestational hypertension and diabetes, parity, infant sex, gestational age at delivery, and total number of prenatal care visits;  **^§^**Poisson regression models were adjusted for maternal age, race/ethnicity, educational levels, marital status, maternal pre-pregnancy body mass index, parity, infant sex, gestational age, and total number of prenatal care visits;  ^#^Imputation by the fully conditional specification method;  **^†^** Only for boys;  ^⁑^ Any outcome was defined as having any one of congenital diaphragmatic hernia, gastroschisis, limb reduction defect, cleft lip with or without cleft palate, cleft palate alone, and hypospadias (only added for the smoking before pregnancy or smoking in the first trimester). | | | | | | | | | | | |
